# Environmental filtering of bacterial functional diversity along an aridity gradient

**DOI:** 10.1038/s41598-018-37565-9

**Published:** 2019-01-29

**Authors:** Ho-Kyung Song, Yu Shi, Teng Yang, Haiyan Chu, Jin-Sheng He, Hyoki Kim, Piotr Jablonski, Jonathan M. Adams

**Affiliations:** 1 Laboratory of Behavioral Ecology and Evolution, Department of Biological Sciences, Seoul National University, Seoul, 151-742 South Korea; 2 State Key Laboratory of Soil and Sustainable Agriculture, Institute of Soil Science, Chinese Academy of Sciences, East Beijing Road 71, Nanjing, 210008 China; 3 Department of Ecology, College of Urban and Environmental Sciences, Peking University, 5 Yiheyuan Road, Beijing, 100871 China; 4 Celemics Inc., 19F, Bldg. A, BYC High city, 131, Gasandigital 1-ro, Gwumcheon-gu, Seoul, 153-718 Korea; 5 Museum and Institute of Zoology, Polish Academy of Sciences, Wilcza 64, Warsaw, Poland; 6 School of Water, Energy and Environment, Cranfield University, Cranfield, MK43 0AL UK

## Abstract

Studying how metagenome composition and diversity varies along environmental gradients may improve understanding of the general principles of community and ecosystem structuring. We studied soil bacterial metagenomes along a precipitation gradient on the eastern Tibetan Plateau, varying between 500 mm and 60 mm mean annual precipitation (MAP). We found that lower MAP was strongly associated with reduced functional diversity of bacterial genes. It appears that extreme environmental conditions associated with aridity constrain the diversity of functional strategies present in soil biota – analogous to broad scale patterns found in plant functional diversity along environmental gradients. In terms of specific functions, more extreme arid conditions were also associated with increased relative abundance of genes related to dormancy and osmoprotectants. Decreased relative abundance of genes related to antibiotic resistance and virulence in more arid conditions suggests reduced intensity of biotic interaction under extreme physiological conditions. These trends parallel those seen in earlier, more preliminary comparisons of metagenomes across biomes.

## Introduction

Soil metagenomes can potentially reveal patterns of variation in functionality of soil biota – and from these the general ecological patterns of community structure and ecosystem function^1^. The search for community-level traits in metagenomes is based upon genes considered to code for these traits, rather than observation of the traits themselves, which would often be difficult to observe and quantify within the soil biota.

Our primary interest in this study was the effect of an environmental stress gradient on the functional diversity of the soil biota. Theory and observation of larger organisms, notably plants, has both predicted and observed broad scale gradients in the diversity of functional traits along climatic stress gradients^2–4^. The authors of those studies suggested that extreme physiological conditions and reduced energy budgets for organisms constrain the number of different functional strategies which are viable in a particular environment. We were interested in testing whether similar rules also hold true for soil biota, with a more extreme environment being associated with decreased diversity of functional traits.

There have been various studies on the effect of climatic aridity on overall microbial community structure^5,6^. For example, Maestre *et al*.^5^ studied microbial community structure in a wide range of global drylands. They found that microbial diversity decreased along a gradient of increasing aridity, and that relative abundances of major groups of bacteria and fungi are correlated with the degree of aridity. However, the effects of aridity stress on relative abundance of traits and overall functional trait diversity in soil biota are largely unknown.

In this study, we aimed to use whole metagenome shotgun sequencing of soil DNA to understand how a gradient in aridity - and aridity-associated stress gradients such as salinity - may affect functional diversity of genes in the soil bacterial community, and the relative abundances of genes associated with particular functional traits. Aridity, together with temperature, has long been regarded as one of the ultimate driving factors behind ecosystem variation^7^. We chose the Tibetan Plateau as our study area because it presents a strong aridity gradient under similar mean annual temperature conditions.

We expected functional gene diversity to be more constrained along a gradient of increasing aridity, and reduced functional diversity to be closely associated with reduced OTU (operational taxonomic unit) diversity. Extreme conditions may require a larger proportion of an organism’s energy and other resources to be diverted into homeostasis (e.g. against extreme osmotic conditions, extreme pH, lack of water etc.)^8,9^. This may preclude other functional traits which could otherwise enhance survival (e.g. through additional strategies of nutrient acquisition, mutualism, or interference competition) or allow occupation of a wider range of different types of niches.

We also expected that there would be significant differences in functional gene composition along an aridity gradient, in terms of which gene types are relatively more common in the metagenome. In terms of the prevalence of particular functional traits along an aridity gradient (defined in terms of a spatial climate gradient from higher precipitation to lower precipitation), we hypothesized the following trends based on general ecological principles.

### The relative abundance of genes related to stress responses will increase towards more arid conditions

Desert environments are often regarded as ‘stressful’ because of high disturbance rates through wind erosion, high salinity, low moisture content, low nutrient concentrations, and low primary productivity. We thus expected that bacteria and other micro-organisms will cope with (a) water deficiency by accumulating protectant solutes^10–12^, and (b) with oxidative stress (due to high radiation) by producing antioxidants^13^. Bacteria will also (c) more frequently possess dormancy and sporulation mechanisms to cope with extreme conditions^14^, when their cells cannot sustain basic metabolism.

### The relative abundance of genes related to competitive traits will decrease towards more arid conditions, whereas the relative abundance of housekeeping genes will increase

In ecology, it is generally considered that in extreme environments, the physiological costs and functional constraints on organisms leave relatively little of their resources for interference competition^15,16^. Additionally, the slow rates of growth and population increase in extreme environments - relative to rates of disturbance - are seen as resulting in less intense competition^17^. Thus, we anticipated that there would be relatively low abundances of genes relating to competitive traits, for example: (a) production of secondary metabolites and (b) antibiotic resistance^18,19^. Instead, the core housekeeping genes, for example (c) genes related to cell division, (d) DNA metabolism, or (e) protein metabolism, were expected to play a relatively more important role in maintaining basic metabolism of bacterial cells for survival or reproduction^20^.

## Results

After quality control, the total number of shotgun metagenome sequences in the 29 samples was 48,371,589 ranging from 1,046,994 to 2,274,876 per sample. Among these qualified sequences, 9,686,708 sequences, ranging from 205,826 to 461,729 per sample, were successfully annotated with the e-value cutoff of 10^−5^, minimum % identity of 60%, and minimum alignment length cutoff as 15 bp.

Also, after quality control, the total number of 16S rRNA amplicon sequences from the 29 samples was 190,058. The number of sequence reads in each sample ranged from 4,036 to 10,400. The most abundant phylum was Actinobacteria (32.92%) followed by Proteobacteria (28.00%), Bacteroidetes (7.84%), Acidobacteria (5.97%), and Firmicutes (2.87%) (Fig. S1) Jing *et al*.^21^. After subsampling into 4,000 reads, the total number of OTUs in the 29 samples was 35,770 ranging from 1,174 to 2,456 per sample.

### Functional gene composition along an aridity gradient

**Figure 1:**
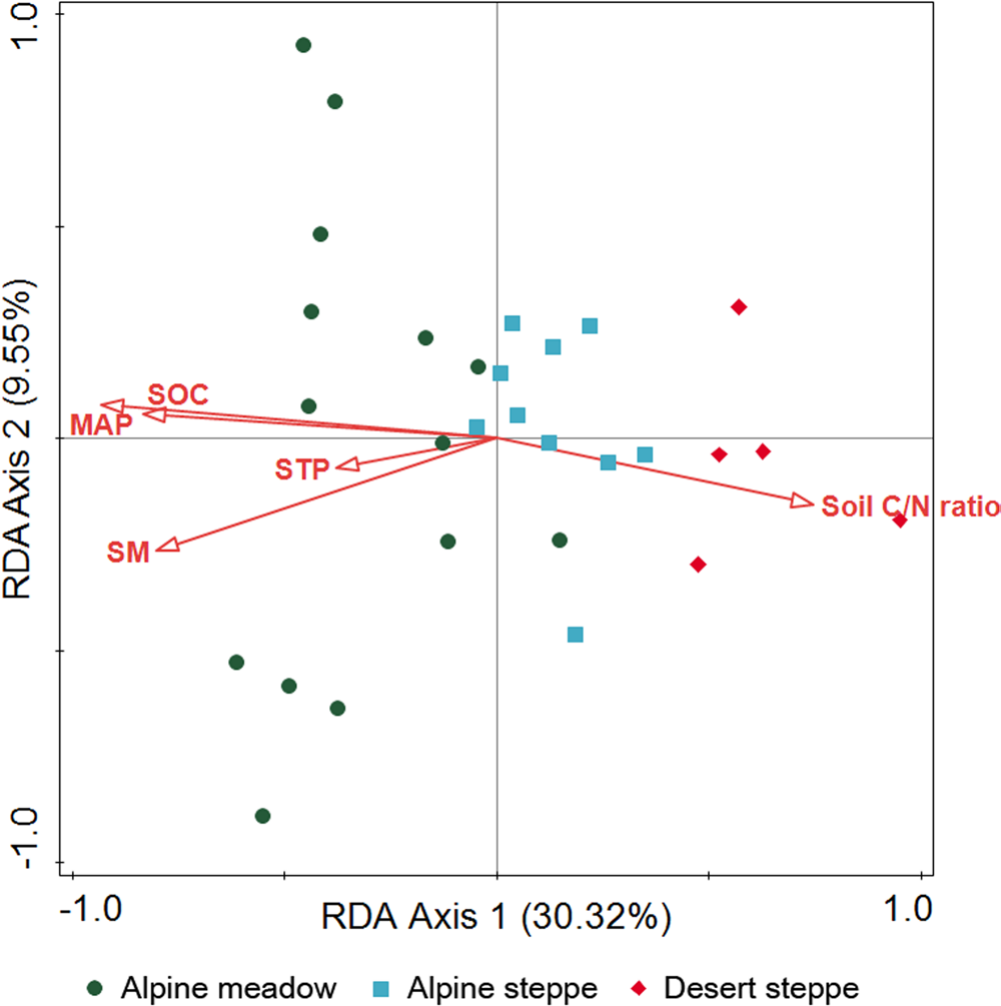
RDA result (Subsystem Level 3) showing sample distribution based on bacterial functional gene composition along the environmental gradient in Tibet. Vegetation types are indicated, with MAP of alpine meadow > alpine steppe > desert steppe. MAP: mean annual precipitation (mm), SM: soil moisture (g/g dried soil), SOC: soil organic carbon (%), STP: soil total phosphorous (%).

**Figure 2:**
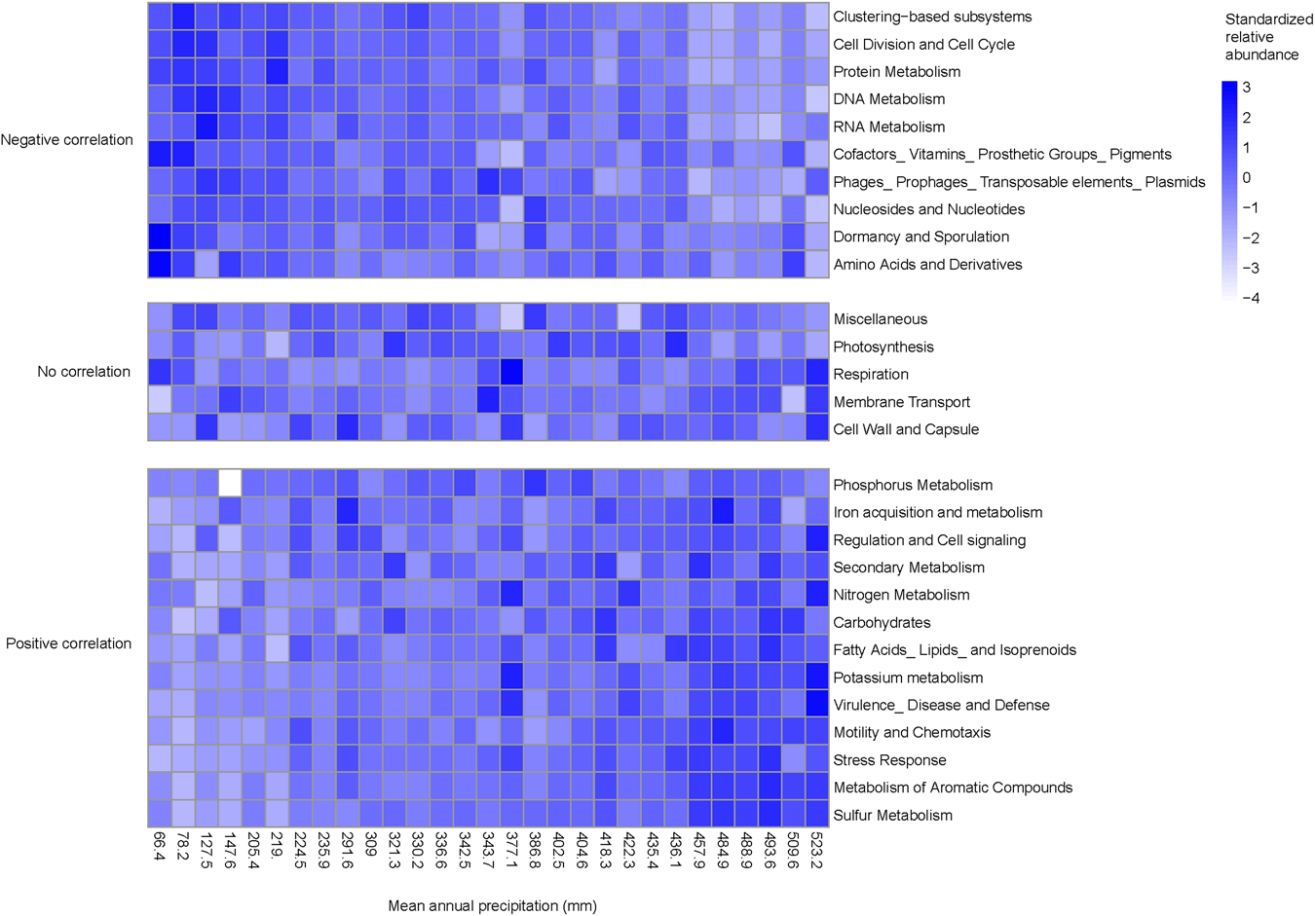
Heatmap of Z-score transformed relative abundance of Subsystem Level 1 genes in samples along the gradient of mean annual precipitation (mm).

The RDA result of Subsystem Level 3 genes showed that mean annual precipitation was the best predictor for functional gene composition, alone explaining 26.7% of total variation (Fig. 1 and Table S1; results of Subsystem Level 4 genes are shown in Fig. S2 and Table S2). A heatmap of standardized Subsystem Level 1 gene relative abundance along the mean annual precipitation gradient displays gene categories variously having significant positive correlation (p < 0.05, Pearson’s correlation coefficient was automatically larger than 0.52), negative correlation (p < 0.05, Pearson’s correlation coefficient was automatically lower than −0.44) or no correlation (p > 0.05) (Fig. 2). As mean annual precipitation (MAP) increased, the relative abundance of genes related to cell division and cell cycling, dormancy and sporulation, DNA metabolism, RNA metabolism, protein metabolism, nucleotides and nucleosides, decreased. Relative abundance of genes related to nutrient cycling (sulfur metabolism, potassium metabolism, carbohydrates, nitrogen metabolism, iron acquisition and metabolism, phosphorous metabolism, fatty acids) and metabolism of aromatic compounds increased with increasing MAP. The relative abundance of genes related to motility, regulation and cell signaling and secondary metabolism also increased. Under the regulation and cell signaling gene category, only one Subsystem level 2 gene category, “proteolytic pathway”, showed a significant positive correlation in relative abundance with MAP (correlation coefficient of 0.535, p = 0.003).

Genes related to osmotic stress (under the stress response category) that have significant correlation in their relative abundance with MAP are shown in a more detail in Table S3. Overall the relative abundance of genes related to osmotic stress (Subsystem Level 2) had significant positive correlation with MAP. The relative abundance of genes related to synthesis of osmoregulatory periplasmic glucans increased as MAP increased, whereas the relative abundance of genes related to production of osmoprotectants (“Betaine biosynthesis from glycine”, “ectoine biosynthesis and regulation”) and osmoprotectant transporters (“osmoprotectant ABC transporter YehZYXW of Enterobacteriales”) decreased. The relative abundance of genes related to protein chaperones also decreased significantly with increasing MAP (correlation coefficient of −0.426, p = 0.0213)

Genes related to oxidative stress (under stress response category) that show significant correlation in their relative abundance with MAP are shown in a more detail in Table S4. Overall, genes related to oxidative stress (Subsystem Level 2) had a significant positive correlation in relative abundance with MAP. The relative abundance of genes related to antioxidants such as glutathione increased as MAP increased. We searched for taxonomic annotation of those genes that have significant positive correlation in their relative abundance with MAP in alpine meadow samples (where MAP is the highest), and found that *Rhodopseudomonas* and *Bradyrhizobium* were the most abundant genera (Fig. S3). We verified that the relative abundance of these two genera increased along with MAP (*Rhodopseudomonas* had a correlation coefficient of 0.620 and p value of 0.000; *Bradyrhizobium* had a correlation coefficient of 0.638 and p value of 0.000), a trend possibly related to increases in plant root biomass and activity with greater MAP.

Since many genes related to antibiotic resistance were grouped together in a very heterogeneous category with genes related to heavy metal resistance under “resistance to antibiotics and toxic compounds (Subsystem Level 2)”, we manually selected genes with annotated labels related to antibiotic resistance and performed correlation analysis. The relative abundance of genes related to antibiotic resistance showed a strong positive correlation with MAP (correlation coefficient = 0.787, p < 0.0001)

### Functional gene diversity vs OTU diversity

#### Alpha diversity

**Figure 3:**
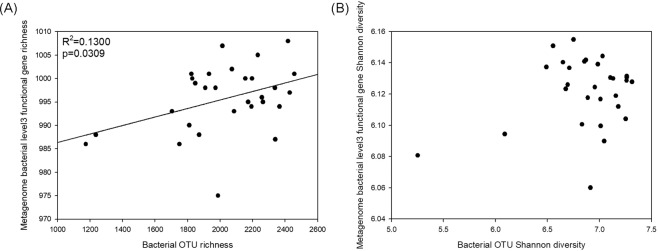
Subsystem Level 3 functional gene richness against bacterial OTU richness. (**A**) Subsystem Level 3 functional gene Shannon diversity against bacterial OTU Shannon diversity. (**B**) Linear regression line was applied only when significant. Presented R-squared value is adjusted R-squared value.

Subsystem Level 3 functional gene richness increased as bacterial OTU richness increased (Fig. 3-A), but Subsystem Level 3 functional gene Shannon diversity showed no significant correlation with OTU Shannon diversity (Fig. 3-B). Subsystem Level 4 functional gene richness had no significant correlation with bacterial OTU richness (Fig. S4-A) and Subsystem Level 4 functional gene Shannon diversity had no significant correlation with OTU Shannon diversity (Fig. S4-B).

#### Beta diversity

OTU and functional gene beta diversity between samples calculated based on Bray-Curtis dissimilarity between sample points showed a significant correlation. (Mantel test of 999 permutations, Pearson correlation, for Subsystem Level 3 genes, Mantel static r = 0.8376, p < 0.001; for Subsystem Level 4 genes, Mantel static r = 0.8549, p < 0.001).

### Functional gene diversity along aridity gradients

#### Alpha diversity

**Figure 4:**
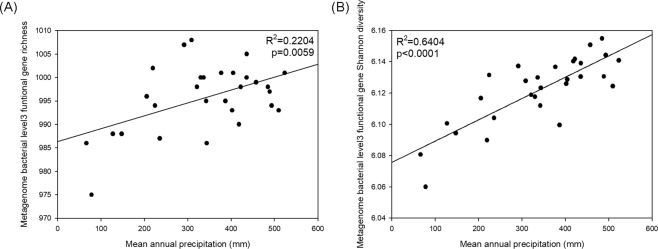
Subsystem Level 3 functional gene richness against mean annual precipitation. (**A**) Subsystem Level 3 functional gene Shannon diversity against mean annual precipitation. (**B**) Linear regression line was applied. Presented R-squared value is adjusted R-squared value.

Both richness and Shannon diversity of functional genes decreased towards more arid conditions (Fig. 4; Subsystem Level 4 gene result is shown in Fig. S5). The correlation was stronger than that between OTU richness/Shannon diversity and aridity (Fig. S6). When both OTU richness and MAP were included as explanatory variables in a linear regression model predicting functional gene richness, only MAP was picked as a significant variable (p value for OTU richness: 0.129, p value for MAP: 0.0239).

Since pH dominates many aspects of soil ecology^1,22^, we also tested whether there is any correlation between pH and OTU/functional gene diversity that might better explain the variation seen along the aridity gradient. The regression analysis of bacterial OTU diversity against pH showed a non-significant result (Fig. S7), but pH was able to explain 22% of variation in functional gene diversity (Fig. S8). Compared to MAP, which explained 64% of variation in functional gene diversity, far less variation was explained by pH. When we put both MAP and pH as explanatory variables in a linear regression model, only MAP was selected as a significant variable (p value of MAP was 8.08 * 10^−6^ whereas p value of pH was 0.688).

#### Beta diversity

**Figure 5:**
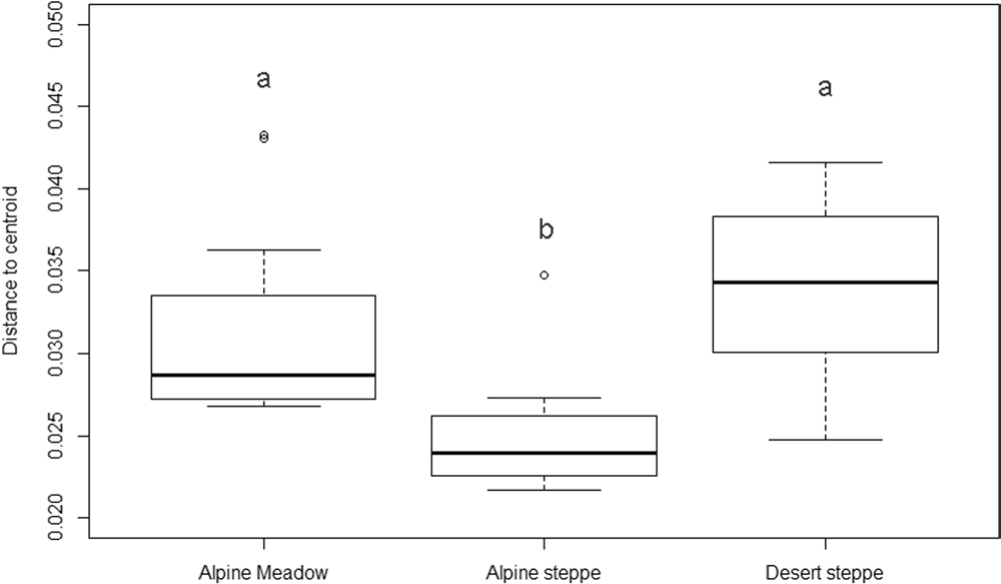
Functional beta diversity of bacterial functional genes, amongst samples from each vegetation type calculated based on Bray-Curtis dissimilarity of Subsystem Level 3 genes from group centroid. Alphabet denotes posthoc test result of Tukey’s HSD.

We grouped samples by vegetation type (which is broadly representative of sections of the aridity gradient) and compared beta diversity calculated based on Bray-Curtis dissimilarity from group centroid of functional gene composition (Fig. 5). In contrast to alpha diversity, although the geographical distance from each group centroid does not differ much between groups (Fig. S9), averaged beta diversity was greatest in desert steppe samples, which had lowest average mean annual precipitation (128 mm) (Fig. 5; Subsystem Level 4 gene result is shown in Fig. S10).

## Discussion

In this study, we show in a field based observational study how total functional diversity and the relative abundance of gene categories varies with a broad scale aridity trend. Previously, such differences have been studied mostly by culturing individual bacterial groups isolated from desert environments^23^. Our findings mostly parallel those seen in earlier studies of arid environments such as Fierer *et al*.^24^ - which demonstrated functional differences of microbial community in desert biome compared to non-desert biomes in a worldwide scale - or Tripathi *et al*.^22^, which compared the functional profile of microbes in arid, semi-arid, Mediterranean, and humid Mediterranean environments in Israel. However, our study is distinguished from these two other studies in scale, geographical location, and sampling methods (e.g. sampling at points scattered along a gradient instead of discrete sampling clusters).

### Functional gene diversity against MAP or OTU diversity

As hypothesized, bacterial functional gene diversity decreased along the gradient of increasing aridity. It appears that more arid conditions may indeed constrain the number of traits that are viable and hence retained in the community, as discussed by Kleidon *et al*.^3^, and Kleidon and Mooney^2^ for plants. Similarly, Mallen-Cooper *et al*.^25^ found in their study on biocrust that aridity had strong and significantly negative effects on functional diversity, suggesting that a reduced number of functional guilds of microbes are able to survive in arid environments. Our findings appear to strengthen the general principle, applicable to both microorganisms and macroorganisms, that extreme environments constrain functional diversity.

We found that the relationship between bacterial functional gene diversity and aridity (Fig. 4) was much stronger than the relationship between OTU diversity and aridity (Fig. S6). It seems that the bacterial species found towards the moister end of the gradient in alpine meadow or alpine steppe samples may be more specialized ecologically (for example, in having genes related to degradation of complex nutrient sources) and that there may be lower functional redundancy in these samples compared to desert steppe samples. It is widely known that reduced taxonomic diversity does not necessarily correlate with reduced functional diversity due to functional redundancy^26,27^. It seems that functional redundancy, which indicates that the same ecological function can be carried out by multiple bacterial species^26^, should be considered as separate from the relationship between taxonomic diversity and functional diversity.

Intriguingly, functional *beta* diversity was greatest in the desert samples. For macro-organisms, there is some evidence that the final community structure in extreme conditions is spatially more uniform than in mesic, productive conditions^28,29^. This may not be applicable to microbes, since microbes exist in much smaller microsites than macro-organisms. Greater beta-diversity of soil microbes in more arid environments in our study might be derived by a habitat heterogeneity effect, due to stronger influence of substrate rock in an arid environment where weathering has exerted a weaker effect on soil chemistry^30^. It might also be due to an ‘island of fertility’ effect whereby locally more resource-rich environments could produce greater patchiness in bacterial community traits^31,32^.

### Functional gene relative abundances along aridity gradient

#### The relative abundance of genes related to stress responses will increase along a gradient of increasing aridity

The overall assemblage of genes related to stress responses showed an unexpected pattern in Fierer *et al*.^24^ and Tripathi *et al*.^33^ because their relative abundance was lower in arid conditions, compared to mesic conditions. We also found in our study that overall relative abundance of genes related to stress responses decreased along a gradient of increasing aridity.

However, when we looked in more detail within these categories down to Subsystem Level 3, we were able to find some reasons for these unexpected patterns. In our study, the relative abundance of the general category of genes related to bacterial stress response (Subsystem Level 1) and genes related to bacterial osmotic stress (Subsystem Level 2) decreased in relative abundance as aridity increased – but within the overall category of genes related to osmotic stress response, this trend was dominated by bacterial genes relating to “Synthesis of osmoregulated periplasmic glucans”, which are produced when there is excessive water^34^. This might then explain why moister climates were associated with greater relative abundance of these genes, due to increased average soil water content, and the frequency of bursts of very high soil water content. However, the relative abundance of the subcategories of bacterial genes related to production of osmoprotectants such as betain or ectoine increased with increasing aridity as would be expected^10,11^. Hence, investigating this hypothesis seems to be partly a case of studying the correct subset of stress related genes in relation to aridity gradients. Also, the relative abundance of bacterial genes relating to “protein chaperones” increased with increasing aridity, as expected. In these senses, then, the hypothesis was supported by the results.

However, the relative abundance of genes related to oxidative stress did not show the expected pattern even when we investigated Subsystem Level 3 genes. The relative abundance of core genes related to oxidative stress response (e.g. production of glutathione) was higher in the samples with increased MAP. This might be due to greater abundance of *Bradyrhizobium* and *Rhodopseudomnas* in these samples. These two genera belong to the family Bradyrhizobiae which play a significant role in global nitrogen cycles^35^. Both genera possess nitrogenase which is a key enzyme in nitrogen fixation and is extremely oxygen sensitive^36^. There are numerous papers reporting the importance of oxidative stress response of these taxa^37,38^. This may related to greater plant root biomass and activity in alpine meadows, as plant roots may support diazotrophs in the rhizosphere^39,40^.

Also, we found the relative abundance of genes related to dormancy to be higher in more arid conditions in all three published studies that have studied metagenomes in arid and non-arid environments (our study, Fierer *et al*.^24^, and Tripathi *et al*.^33^). Dormancy is a well-known mechanism for microbes to cope with stressful conditions, helping bacterial species to conserve energy until a brief window of time in which to grow, feed and reproduce comes^14^. High relative abundances of these genes in the arid conditions found in our study and others place emphasis on the role of dormancy as a survival strategy of bacterial species in extreme conditions.

#### The relative abundance of genes related to competitive traits will be decrease with increasing aridity whereas the relative abundance of housekeeping genes will increase with increasing aridity

The relative abundance of genes related to competitive traits - for example genes classified as related to “virulence, disease, and defense” (including genes related to antibiotic resistance), and genes related to secondary metabolism or proteolytic pathways - was also found to be higher in the least arid environments in all the three studies. A similar pattern has also been found by Goberna *et al*. (2014)^41^. They compared vegetated patches with open unvegetated patches (considered as indicative of stressful conditions) in a semi-arid environment. They found competitive traits to be consistently more abundant in the less stressful, plant-covered patches. Le *et al*.^42^ also found that the types of defense related genes in their hypolith metagenomes clustered more closely with the desert samples of Fierer *et al*.^24^ rather than their non-desert samples, supporting their expectations. As aridity increases, cell density decreases^43^, and this may also be expected to decrease selection for competitive traits, by analogy with the stress-selected strategy of Grime for plants^15^

Also, we found the relative abundance of housekeeping genes (genes related to DNA metabolism, RNA metabolism, and protein metabolism, cell division and cell cycle) increased with increasing aridity – though this may simply reflect the lack of the other ‘extra’ genes for competitive traits rather than any particularly strong selection for housekeeping genes themselves. This pattern was also found in Fierer *et al*.^24^ or Tripathi *et al*.^33^. Koo *et al*.^20^ also reported high abundances of genes related to DNA replication, protein folding, protein biosynthesis, and transcription termination protein in extreme cold conditions. Though it was not discussed in detail by Fierer *et al*.^24^ or Tripathi *et al*.^33^, it seems these genes are needed for bacterial species to maintain steady-state of cellular metabolism, growth, or cell division.

We were also able to find additional interesting trends that had not been hypothesized. For example, that the relative abundance of genes related to nutrient cycling decreased with increasing aridity. High concentrations of nutrients and higher plant biomass in non-arid environments may have selected for a variety of types of nutrient cycling related genes. For instance, aromatic compounds are naturally produced by many macro-organisms, an abundant example being the lignin of plants^44^, and these may have selected for bacterial species carrying genes which can allow them to utilize aromatics as their nutrient source.

Also, we found the relative abundance of genes related to motility decreased with increasing aridity. In plant ecology, the average spatial dispersal distance of seeds in the plant community decreases towards more arid conditions, due to differences in investment in dispersal traits by plants^16,45^. Favorable growing conditions are ephemeral and rare in the desert landscape, and as a result plant seeds in deserts have evolved to stay close to the mother plant and in a dormant state until rain occurs, rather than disperse across the landscape to find favorable conditions^45^. For bacteria too, a more conservative strategy of remaining in place in favorable microsites may be ecologically selected in an arid environment. The genes involved in motility which varied in their relative abundance along the aridity gradient were mostly those related to flagellae: motility in the bacterial world most often depends upon flagellae, which represent an active form of movement. It is also necessary to bear in mind that as flagallae require water surrounding the cells, these might be of less ecological benefit within a soil that is dry most of the time.

## Conclusions

Overall, our findings in this study suggest certain predictable functional patterns in the microbial world, in response to environmental gradients. A striking trend towards decreased functional gene diversity resembles the trends in functional trait diversity seen in macro-organisms such as plants. The trends in relative abundance of particular categories of genes – for example decreased relative abundance of genes associated with competitive traits along aridity gradient - also imply certain basic principles in common between the world of macro-organisms and the world of micro-organisms. It is interesting that most of the trends we found in this study parallel those found by other recent studies on arid-land microbial communities, reinforcing the view that these may be general trends^24,33^.

As a basis for future work, it is important to recognize that the bacterial functional diversity as recognized here is based only on gene diversity, which is not necessarily the same as the phenotypic functional diversity of the whole bacterial community. For instance, some bacterial OTUs, while present, may be relatively more active^46^ such that their abundances do not reflect their functional importance in the community. Furthermore, some gene functional types may be misassigned by MG-RAST (metagenomics Rapid Annotation using Subsystem Technology)^47^, which is an ongoing project to categorize the huge diversity of naturally occurring genes, and is limited by deducing function from homologues which have already been studied. It would be informative to directly study soil catabolic diversity^48^ and other measures of soil activity, as an additional and more direct way of understanding the full functional trait diversity of these soils and how it varies.

It is also possible that the functional gene profile of these soils is significantly altered by a legacy of ‘old’ DNA from dead or lysed cells^49^. Whether this old DNA negates the relevance of what is seen is a moot point, as it represents a time-integrated view of total community gene diversity rather than a snapshot in time. In any case, it is important to realize that old DNA would only be expected to add to the gene diversity of the desert soils, where DNA may be preserved for longer due to slower turnover rates – yet the more arid soils in our gradient still have lower functional gene diversity. Perhaps, without the signal of old DNA, their gene diversity would be even lower compared to that of moister climate soil. Additionally, since relative abundance of genes in samples was compared, an observed pattern might be due to selection against other genes rather than selection for the particular gene functions that become more abundant in one part of the gradient.

It would also be interesting to study whether other apparent ‘stress’ gradients (e.g. gradients towards in salinity, heat, and concentrations of chemical poisons) are associated with reduced gene functional diversity towards the more extreme end of the gradient. Additionally, a systematic global comparison of metagenomes along broader aridity gradients – expanding on the fairly small number of samples in the preliminary scale study by Fierer *et al*.^24^ would be rewarding in understanding the true extent and scale of these trends, particularly whether these trends actually differ as a result of idiosyncratic factors from one geographical region to another.

## Materials and Methods

### Soil sampling and DNA extraction

We collected soil sample from 29 grassland sites across in Tibetan plateau (Fig. S11). We sampled during the growing season in July-August. The survey area encompassed a wide range of climatic and edaphic conditions (mean annual precipitation range from 66 mm to 523 mm, pH range from 6.0 to 9.1, soil total carbon range from 1.12% to 15.22%). Detailed information is provided in Supplementary Tables S5 and S6. Find Supplementary Information provided as a separate document for methodological details used for measurement of each environmental variable. In each site, seven randomly located 0–5 cm depth soil cores (each 5 cm diameter) were collected across a single 1 square meter quadrat (m^2^). The seven individual soil core samples were placed in one bag and thoroughly mixed to homogenize them – making one sample from which the DNA for a single metagenome was obtained. DNA was extracted from 0.5 g of soil in each sample using a FastDNA Spin kit (Bio 101, Carlsbad, CA, USA), following the manufacturer’s instructions and stored at −40 °C.

### 16S rRNA gene analysis using amplicon sequencing and sequence processing

Bacterial community composition data were obtained from Jing *et al*.^21^ where the same sample cores we analysed here by whole genome sequencing were used for amplicon sequencing. For assessment of bacterial community composition, V4-V5 regions of bacterial 16S rRNA genes were amplified using universal bacterial primer F515 (5′-GTGCCAGCMGCCGCGG-3′)^50^ and R907 (5′-CCGTCAATTCMTTTRAGTTT-3′)^51^. PCR process included 30 cycles of denaturation (94 °C, 30 s), annealing (55 °C, 30 s), and extension (72 °C, 30 s), followed by final extension (72 °C, 10 min). PCR product was purified with agarose gel DNA purification kit (TaKaRa) and sequenced using a Roche FLX454 pyrosequencing machine (Roche Diagnostics Corp., Branford, CT, USA).

Sequences were processed following the Quantitative Insights into Microbial Ecology pipeline (http://qiime.sourceforge.net/). Low quality sequences (average quality score less than 25) and sequences with ambiguous characters were removed. Sequences with more than 200-bp length were selected for further analysis. Sequences were aligned against the Greengenes database using the NAST algorithm. Sequences were assigned into OTUs with 97% similarity and classified using the Greengenes database. More details can be found in Jing *et al*.^21^ on bacterial 16S rRNA sequence analysis.

### Shotgun metagenome sequencing and sequence processing

Genomic DNA was sheared into 150-bp to 250-bp size fragments and processed for a shot-gun sequencing run. The process included the following steps; end-repair, dA-tailing, adapter ligation and pre-PCR for indexed NGS library. The PCR product were sequenced in Celemics Inc. (Seoul, Korea) with the HiSeq. 2500 platform (Illumina, USA) using 2 × 150 bp paired-end runs. Sequences were uploaded to MG-RAST (metagenomics Rapid Annotation using Subsystem Technology) server for quality control and for annotation^47^ (project accession number: mgp20476). Sequences that had more than 5 low quality (with 15 phred score cutoff) bases were removed. Sequences were annotated using BLAT (the BLAST-like alignment tool) algorithm^52^ against M5NR database^53^, which provides nonredundant integration of many databases. Among the integrated databases, we chose Refseq database^54^ for taxonomic annotation of functional genes and SEED subsystem hierarchy^55^ for functional gene annotation. Sequences were annotated using default settings (maximum e-value cutoff of 10^−5^, minimum % identity cutoff of 60%, and minimum alignment length cutoff as 15 bp). Sequences that failed to be annotated by MG-RAST were discarded for further analysis. About 97% of annotated functional gene sequences on average comprised of bacterial sequences, so we focused only on bacterial functional genes and removed sequences derived from other organisms (archaea, viruses, and eukaryotes).

### Statistical analysis

To calculate the relative abundance of bacterial phyla in each sample, we grouped OTUs belonging to the same phylum and summed the number of sequence reads. The number of sequence reads for each phylum was divided by the total number of bacterial sequence reads in each sample. The relative abundance of functional genes was calculated similarly. To calculate the relative abundance of functional gene categories in each sample, we divided the number of sequence reads assigned to a specific functional gene category with the total number of bacterial functional gene sequence reads in each sample that has been annotated by MG-RAST. We used non-subsampled data for calculating relative abundances.

To find functional gene categories which have significant correlation with precipitation gradients, we performed correlation analysis using the relative abundance of each gene. To provide an intuitive visualization of the relationship between MAP and each gene, we drew a heatmap using “pheatmap” package in R^56^ with z-score transformed relative abundance of functional gene categories.

We subsampled our data for whole the other analysis described from now, including RDA and diversity calculations. 16S rRNA sequence reads were randomly subsampled to 4,000 reads per sample. Shot-gun metagenome sequence reads were randomly subsampled to 205,826 reads per sample.

To find the environmental variables that best explained functional gene composition, we performed redundancy analysis (RDA) using Canonco ver. 5 (Biometrics, Wageningen, The Netherlands). We applied forward selection of environmental variables and the Monte Carlo permutation test with 999 random permutations was used for significance test. All of the environmental variables listed in Table S5 were included in the RDA analysis as explanatory variables. Subsampled sequence reads of Subsystem level 3 functional genes were square-root transformed and used as a response variable in the RDA analysis.

To calculate alpha and beta diversity of functional genes we used subsampled sequence reads of Subsystem level 3 functional genes. We focused on Subsystem Level 3 genes to calculate functional diversity, since Level 1 and Level 2 gene categories are more likely to be too broad to be used as a representative indicator of diversity. Also, the finest level (one level below Level 3, which has been called Subsystem Level 4 in this paper) showed very similar results to Level 3, so we only presented our data based on Level 3 functions in our main text and included Level 4 functions as supplementary files.

To calculate beta diversity of OTUs and functional genes, subsampled sequence reads were square root transformed and the Bray-Curtis dissimilarity between each sample point was calculated. To compare beta diversity of OTUs and functional genes, a Mantel test was performed by using “mantel.test” function in R package “vegan”^57^. To compare beta diversity of functional genes in different vegetation types (alpine meadow, alpine steppe, and desert steppe), subsampled Subsystem level 3 functional gene data were square root transformed and Bray-Curtis dissimilarity from group centroid was calculated using “betadisper” function in R package “vegan”^57^. Beta diversity between groups was compared using analysis of variance (ANOVA).

## Supplementary information

Supplementary information
