# Supplementary material for: Environmental filtering of bacterial functional diversity along an aridity gradient
Source: Sci Rep. 2019 Jan 29;9:866. doi: 10.1038/s41598-018-37565-9 (PMC6351613; doi:10.1038/s41598-018-37565-9)
Supplement: Supplementary file 1 — Supplementary information [file 41598_2018_37565_MOESM1_ESM.docx]

**Supplementary Information**

accompanying the manuscript

**Environmental filtering of bacterial functional diversity along an aridity gradient**

Ho-Kyung Song^1^, Yu Shi^2^, Teng Yang^2^, Jin-Sheng He^3^, Hyoki Kim^4^, Piotr Jablonski^1, 5^, Haiyan Chu^2*,^ Jonathan M. Adams^6^*

1. Laboratory of Behavioral Ecology and Evolution, Department of Biological Sciences, Seoul National University, Seoul 151-742, South Korea
2. State Key Laboratory of Soil and Sustainable Agriculture, Institute of Soil Science, Chinese Academy of Sciences, East Beijing Road 71, Nanjing, 210008, China
3. Department of Ecology, College of Urban and Environmental Sciences, Peking University, 5 Yiheyuan Road, Beijing 100871, China
4. Celemics Inc., 19F, Bldg. A, BYC High city, 131, Gasandigital 1-ro, Gwumcheon-gu, Seoul, 153-718, Korea
5. Museum and Institute of Zoology, Polish Academy of Sciences, Wilcza 64, Warsaw, Poland
6. School of Water, Energy and Environment, Cranfield University, Cranfield, MK43 0AL, UK

*(co-corresponding authors).

**Climatic data collection and assessment of environmental factors**

We compiled mean annual temperature (MAT), mean annual precipitation (MAP) data of each site from the National Meteorological Bureau of China database (Jing et al., 2015). Growing season (from April to August) temperature (GST) and growing season precipitation (GSP) data were compiled from the 1950-2000 records of a global climate database (Hijmans et al., 2005; Ma et al., 2010). Potential Evapotranspiration (PET) data were collected from Global Aridity and PET database of CGIAR-CSI (http://cigar-csi.org) (Yang et al., 2017a). Actual evapotranspiration (AET) was determined by comparing potential evapotranspiration with precipitation plus available water stored in the soil as described in Major (1963) and Kreft and Jetz (2007).

Soil moisture (SM) was measured gravimetrically by drying soils at 105℃ for ~10 hours. Soil pH was measured using a pH meter (Thermo Orion-868) in a 1:5 ratio of fresh soil to deionized water slurry. Soil total carbon (STC) and soil total nitrogen (STN) were measured by combustion of the samples in a CHN elemental analyzer (2400 Ⅱ CHN elemental analyzer, PerkinElmer, Boston, MA, USA). Soil total phosphorous (STP) was measured by molybdenum blue method in conjugation with ultraviolet-visible spectrophotometer (UV-2550, Shimadzu, Kyoto, Japan). Soil CaCO3 was measured using a volumetric calcimeter (Eijkelkamp, Netherland). Soil organic carbon (SOC) was calculated by subtracting soil CaCO3 (SCaCO3) from soil total carbon (STC). The density of SOC, STN, STP, SCaCO3 were calculated in the top 0-5cm soil.

Dissolved organic carbon (DOC), dissolved total nitrogen (DTN), and soil mineral nitrogen were extracted by mixing 10 grams of fresh soil with 50 ml of 0.5 M K2SO4, shaking for an hour, and vacuum filtering through glass fiber filters (Fisher G4, 1.2㎛ pore space). Ammonium nitrogen (NH4+-N) content were assessed colourimetrically by automated segmented flow analysis (Bran+Luebbe AAⅢ, Germany) using salicylate/dichloroisocyanuric acid method. Nitrate nitrogen (NO3^-^-N) content were assessed using the same analysis with cadmium column/sulphanilamide reduction method. DOC and DTN was assessed by TOC-TN analyzer (Shimadzu, Kyoto, Japan). Dissolved organic nitrogen (DOC) was calculated by subtracting NH4^+^-N and NO3^-^-N from DTN (Yang et al., 2017b).

To assess aboveground biomass, % total carbon in aboveground biomass (PTC), % total nitrogen in aboveground biomass (PTN), and % total phosphorous in above ground biomass (PTP), plant communities in each 1-m^2^ plot were surveyed and harvested before soil sampling (Jing et al., 2015). Plant materials were dried at 60℃ for ~12 hours and grinded. PTC, PTN, and PTP was analyzed with the same method used for STC, STN, and STP. To assess root biomass, 4-8 soil cores per 1-m^2^ plot (0-5 cm deep, 7 cm diameter) was collected. To calibrate root biomass, live:dead (56:63) ratio was used.

**References**

Hijmans, R.J., Cameron, S.E., Parra, J.L., Jones, P.G., and Jarvis, A. (2005) Very high resolution interpolated climate surfaces for global land areas. *International Journal of Climatology* **25**: 1965-1978.

Jing, X., Sanders, N.J., Shi, Y., Chu, H., Classen, A.T., Zhao, K. et al. (2015) The links between ecosystem multifunctionality and above- and belowground biodiversity are mediated by climate. **6**: 8159.

Kreft, H., and Jetz, W. (2007) Global patterns and determinants of vascular plant diversity. *Proceedings of the National Academy of Sciences* **104**: 5925-5930.

Ma, W., He, J.-S., Yang, Y., Wang, X., Liang, C., Anwar, M. et al. (2010) Environmental factors covary with plant diversity–productivity relationships among Chinese grassland sites. *Global Ecology and Biogeography* **19**: 233-243.

Major, J. (1963) A Climatic Index to Vascular Plant Activity. *Ecology* **44**: 485-498.

Yang, T., Adams, J.M., Shi, Y., Sun, H., Cheng, L., Zhang, Y., and Chu, H. (2017a) Fungal community assemblages in a high elevation desert environment: Absence of dispersal limitation and edaphic effects in surface soil. *Soil Biology and Biochemistry* **115**: 393-402.

Yang, T., Adams, J.M., Shi, Y., He, J.-s., Jing, X., Chen, L. et al. (2017b) Soil fungal diversity in natural grasslands of the Tibetan Plateau: associations with plant diversity and productivity. *New Phytologist* **215**: 756-765.

**Supplementary Figures**

**
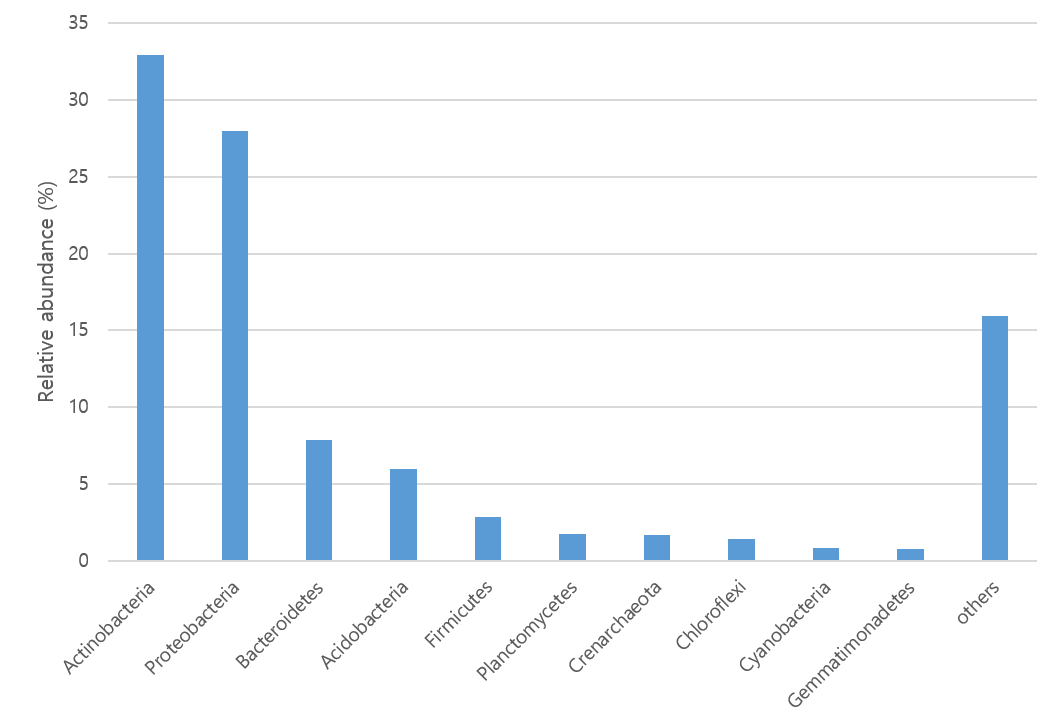
**

**Supplementary Fig. S1.** Phylum breakdown of bacterial OTUs

**
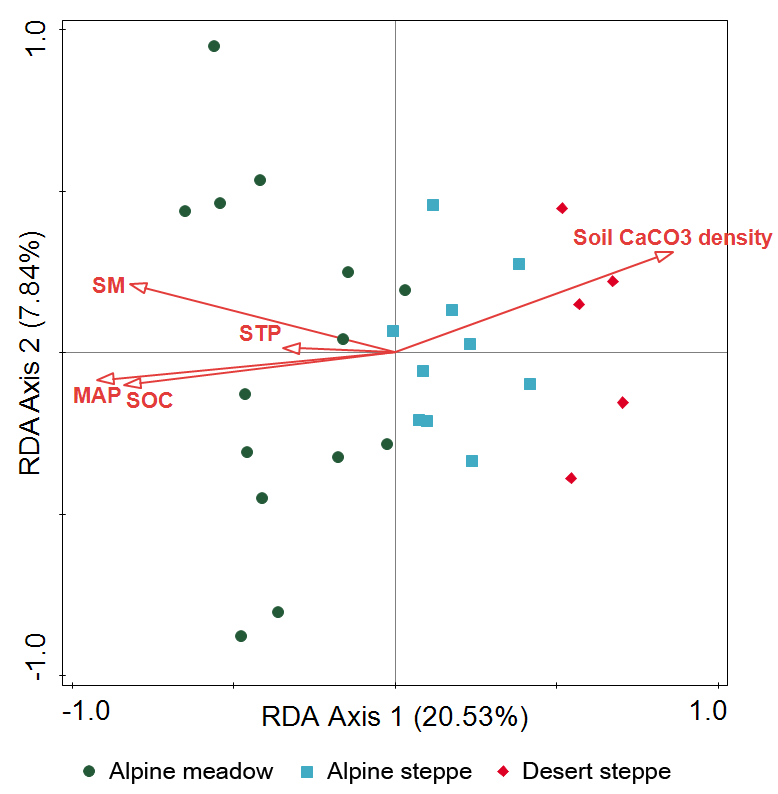
**

**Supplementary Fig. S2.** RDA result (Subsystem Level 4) showing sample distribution based on bacterial functional gene composition along the environmental gradient in Tibet. Vegetation types are indicated, with MAP of alpine meadow>alpine steppe>desert steppe. MAP: mean annual precipitation (mm), SM: soil moisture (g/g dried soil), SOC: soil organic carbon (%), STP: soil total phosphorous (%).

**
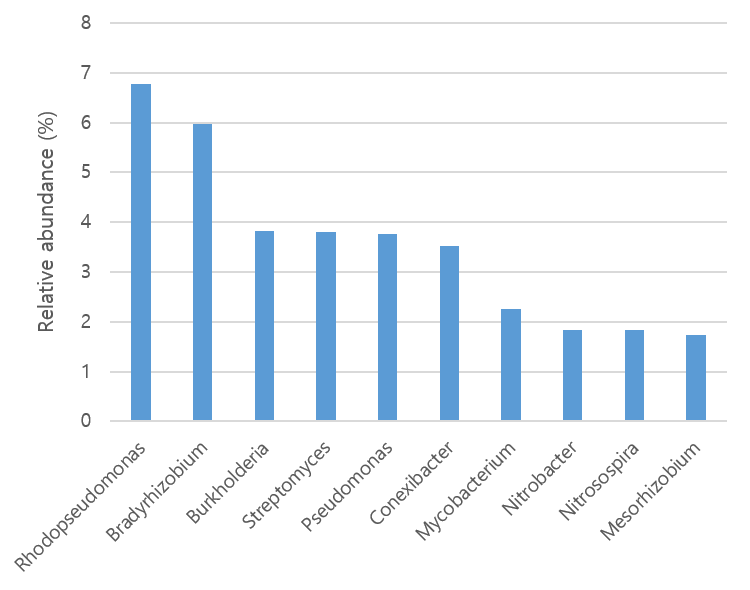
**

**Supplementary Fig. S3.** Taxonomic annotation of genes related to oxidative stress in alpine meadow samples that have significant correlation with MAP

**
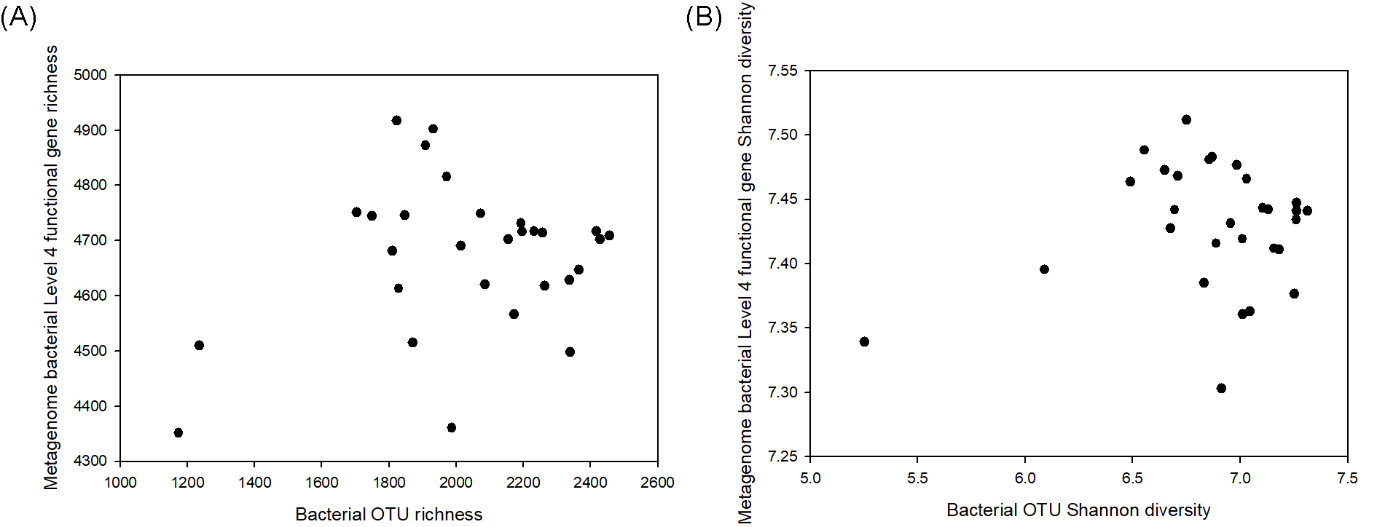
**

**Supplementary Fig. S4.** Subsystem Level 4 functional gene richness against bacterial OTU richness (A). Subsystem Level 4 functional gene Shannon diversity against bacterial OTU Shannon diversity (B). Results not significant (P>0.05).

**
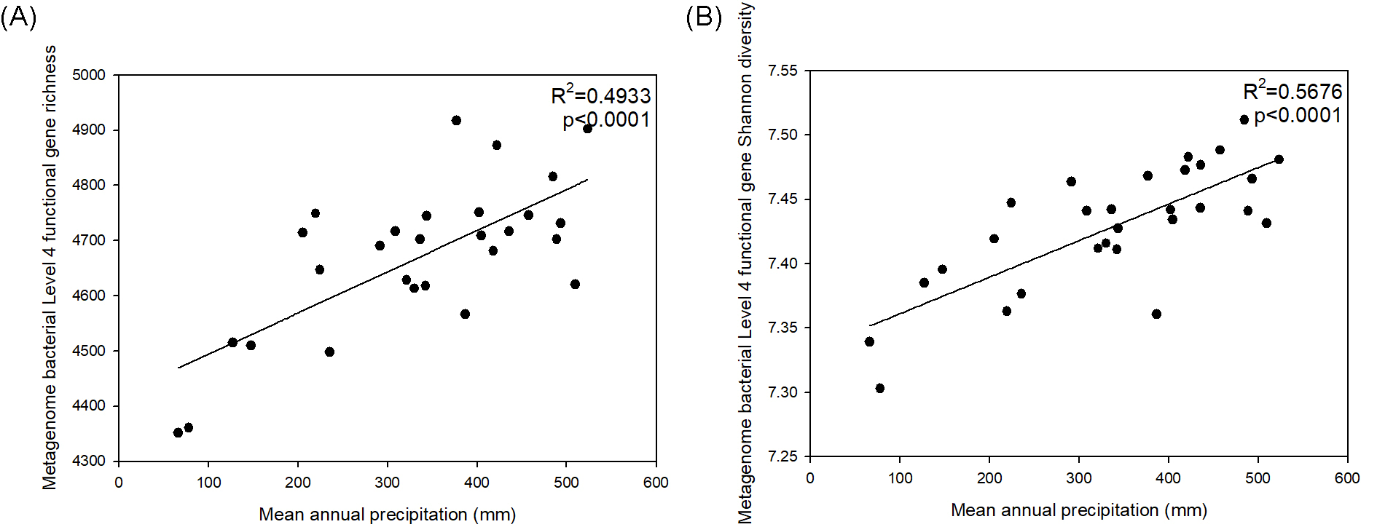
**

**Supplementary Fig. S5.** Subsystem Level 4 functional gene richness against mean annual precipitation (A). Subsystem Level 4 functional gene Shannon diversity against mean annual precipitation (B). Linear regression line was applied. Presented R-squared value is adjusted R-squared value.


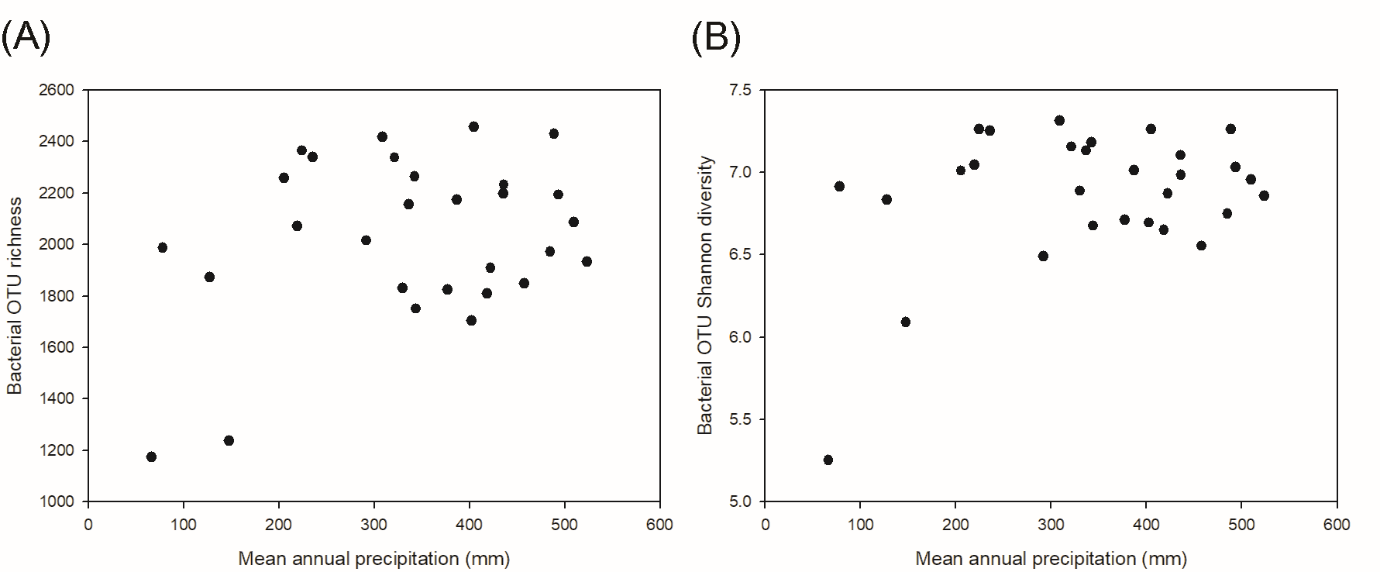


**Supplementary Fig. S6.** Bacterial OTU richness against mean annual precipitation (A). Bacterial OTU Shannon diversity against mean annual precipitation (B). Results not significant (P>0.05).

**
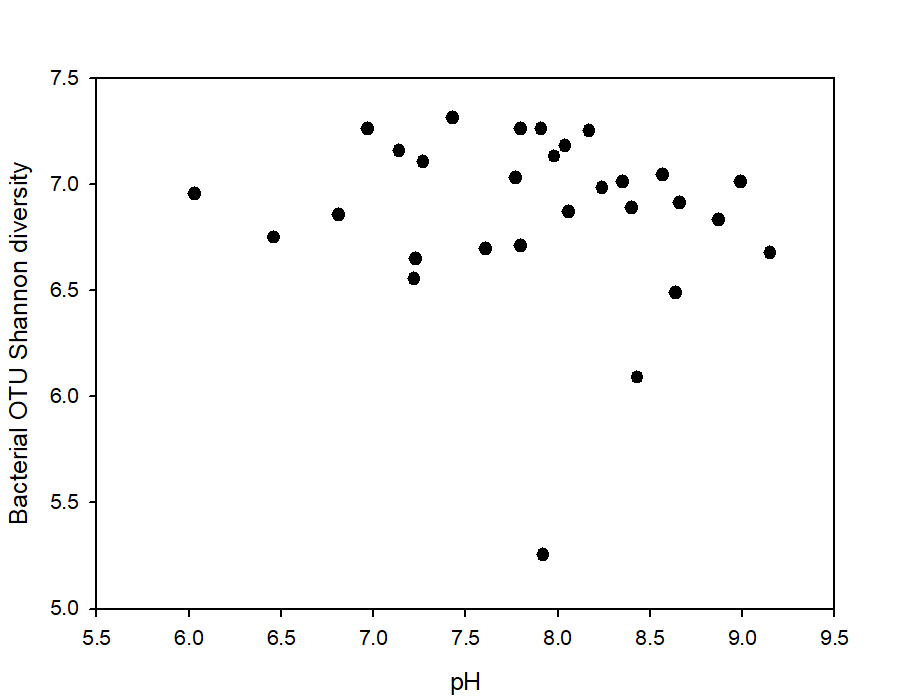
**

**Supplementary Fig. S7**. Bacterial OTU Shannon diversity against pH. Linear regression results not significant.

**
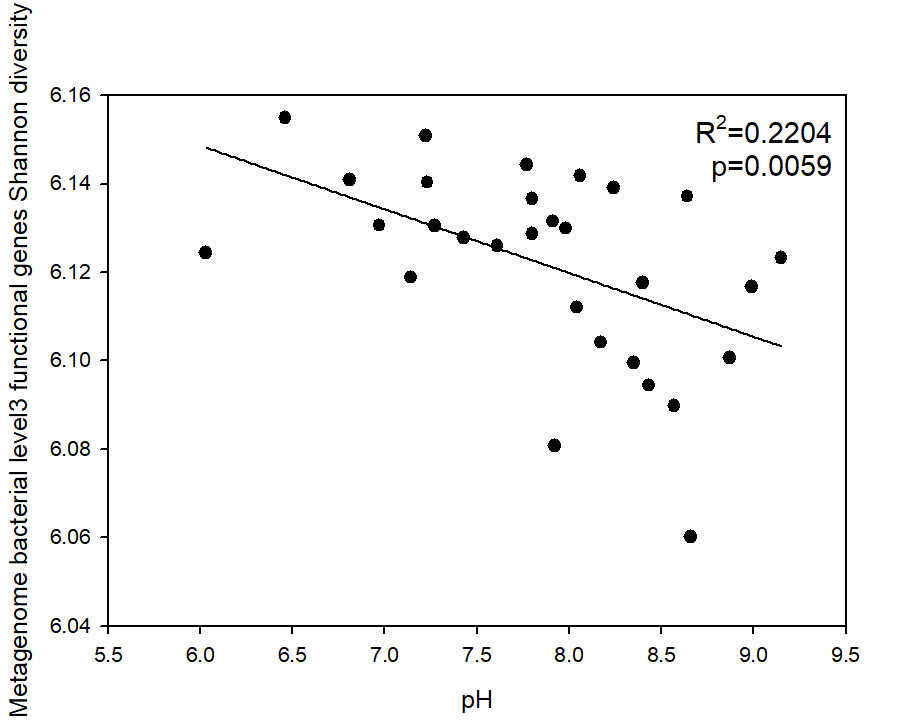
**

**Supplementary Fig. S8.** Metagenome bacterial level 3 functional gene Shannon diversity against pH. Linear regression line was applied. Presented R-squared value is adjusted R-squared value.

**
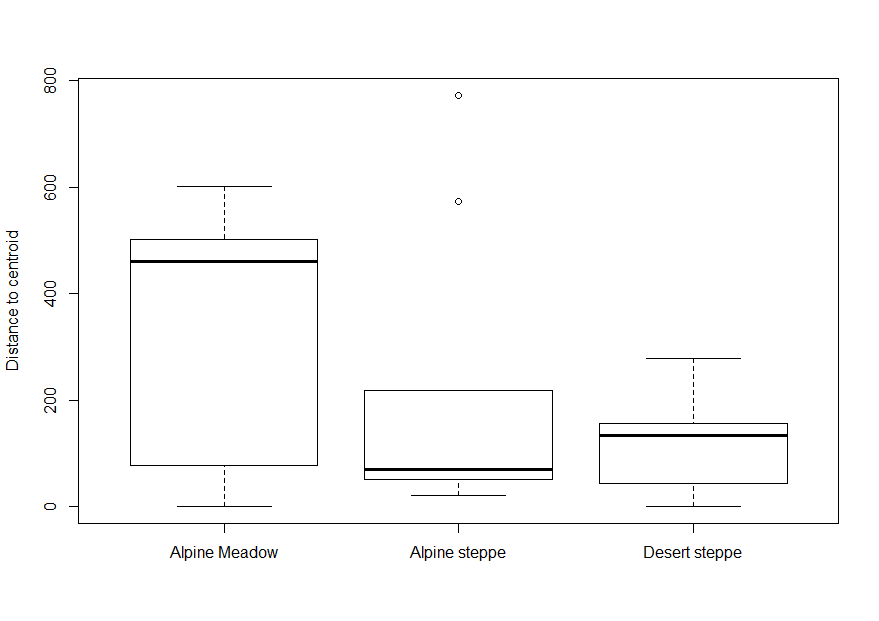
**

**Supplementary Fig. S9.** Spatial distance of each sampling point from group centroid. There was no significant difference between groups (ANOVA, p>0.05).

**
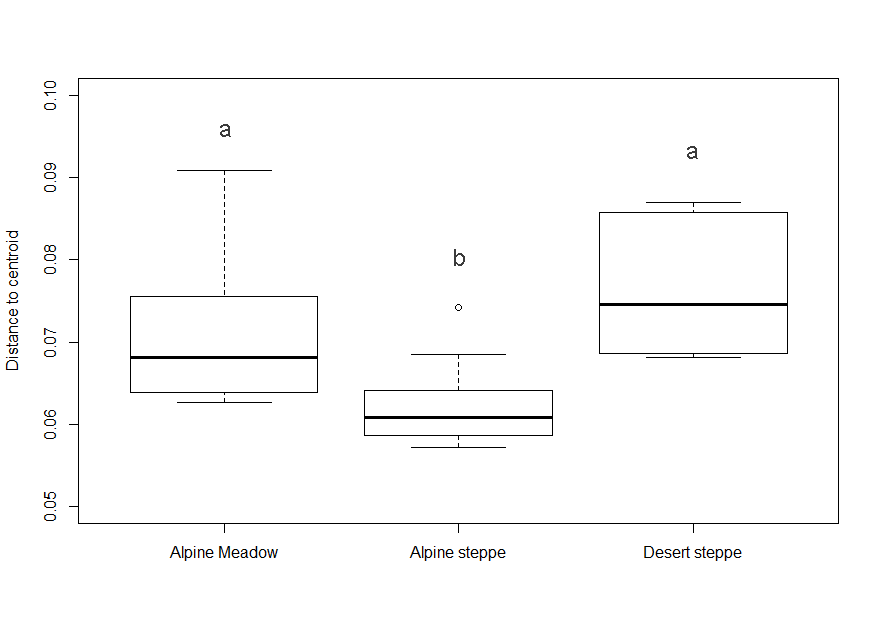
**

**Supplementary Fig. S10.** Functional beta diversity in each vegetation type calculated based on Bray-Curtis dissimilarity of Subsystem Level 4 genes from group centroid. Alphabet denotes posthoc test result of Tukey’s HSD.

**
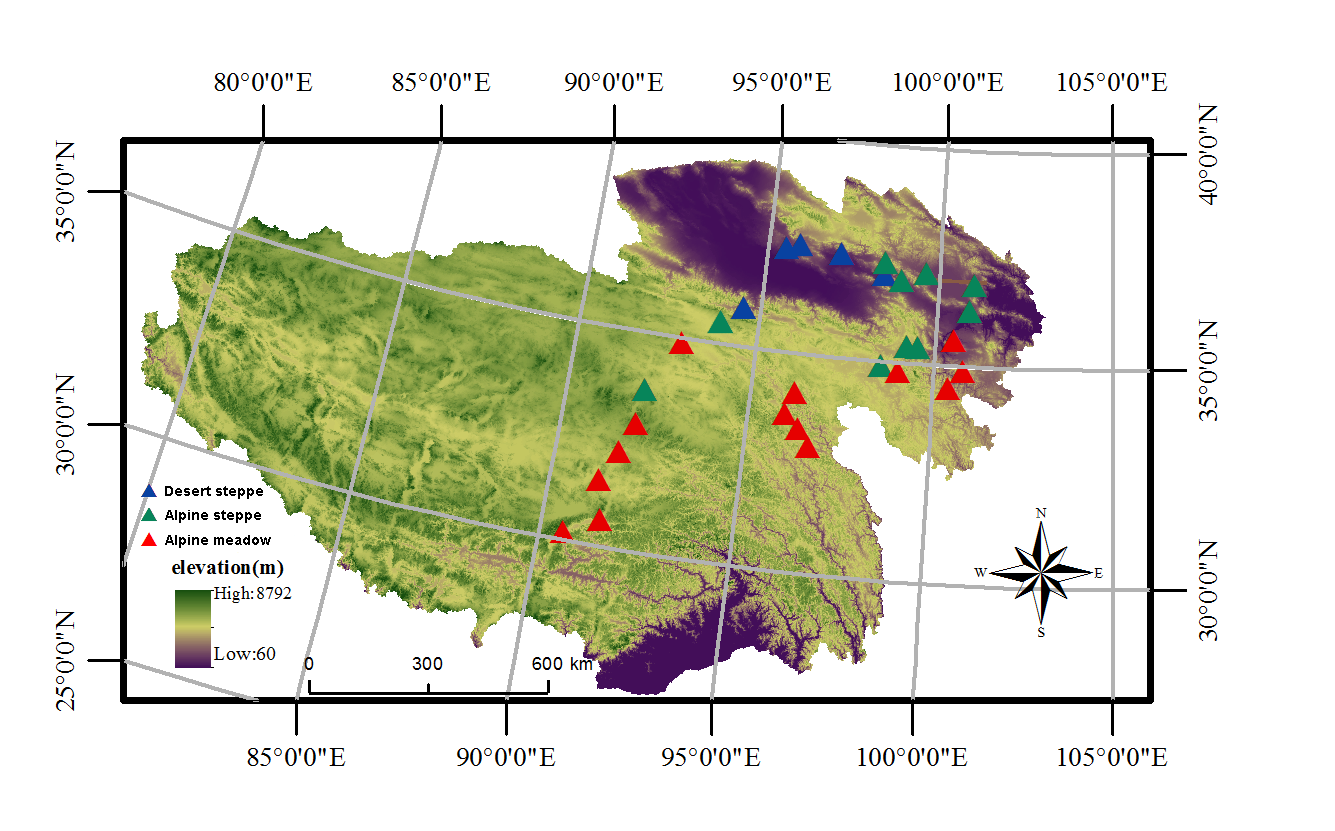
**

**Supplementary Fig. S11.** Map of sample collection site

**Supplementary Tables**

**Table S1.** Forward selection of environmental variables, which best explain variation in functional gene composition (Subsystem Level 3 genes) between samples.

| Environmental variable | Explains % | pseudo-F | P |
| --- | --- | --- | --- |
| Mean annual precipitation (MAP) | 26.7 | 9.8 | 0.001 |
| Soil moisture (SM) | 6.9 | 2.7 | 0.002 |
| Soil organic carbon (SOC) | 7.4 | 3.1 | 0.001 |
| Soil total phosphorous (STP) | 4.1 | 1.8 | 0.013 |
| Soil C:N ratio | 4.0 | 1.8 | 0.03 |

**Table S2.** Forward selection of environmental variables, which best explain variation in functional gene composition (Subsystem Level 4 genes) between samples.

| Environmental variable | Explains % | pseudo-F | P |
| --- | --- | --- | --- |
| Mean annual precipitation (MAP) | 18 | 5.9 | 0.001 |
| Soil moisture (SM) | 5.9 | 2 | 0.001 |
| Soil organic carbon (SOC) | 6.3 | 2.2 | 0.001 |
| Soil total phosphorous (STP) | 4.4 | 1.6 | 0.009 |
| Soil CaCO_3_ (g/m^-2^) | 4.0 | 1.5 | 0.021 |

**Table S3.** Correlation analysis result of genes belonging to the osmotic stress category. Only the genes that have significant correlation in their relative abundance with MAP are shown.

| Subsystem Level 2 | Subsystem Level 3 belonging to osmotic stress category | Relative abundance (%)  (average ± standard deviation) | Correlation coefficient | p value |
| --- | --- | --- | --- | --- |
| Osmotic stress |  | 0.4733 ± 0.0518 | 0.462 | 0.012 |
|  | Betaine biosynthesis from glycine | 0.1678 ± 0.0026 | -0.474 | 0.009 |
|  | Ectoine biosynthesis and regulation | 0.0097 ± 0.0146 | -0.584 | 0.001 |
|  | Osmoprotectant ABC transporter YehZYXW of Enterobacteriales | 0.0014 ± 0.0013 | -0.469 | 0.010 |
|  | Osmoregulation | 0.0509 ± 0.0138 | 0.638 | 0.000 |
|  | Osmotic stress cluster | 0.0043 ± 0.0021 | 0.377 | 0.044 |
|  | Synthesis of osmoregulated periplasmic glucans | 0.1701 ± 0.059 | 0.607 | 0.000 |

**Table S4.** Correlation analysis result of genes belonging to the oxidative stress category. Only the genes that have significant correlation in their relative abundance with MAP are shown.

| Subsystem Level 2 | Subsystem Level 3 belonging to osmotic stress category | Relative abundance (%)  (average ± standard deviation) | Correlation coefficient | p value |
| --- | --- | --- | --- | --- |
| Oxidative stress |  | 1.0330 ± 0.0455 | 0.571 | 0.001 |
|  | Glutathione: Non-redox reactions | 0.0947 ± 0.0179 | 0.702 | 0.000 |
|  | Rubrerythrin | 0.0441 ± 0.0073 | 0.634 | 0.000 |
|  | Oxidative stress | 0.2093 ± 0.0244 | 0.561 | 0.002 |
|  | Protection from Reactive Oxygen Species | 0.1006 ± 0.0146 | 0.555 | 0.002 |
|  | Glutathione: Redox cycle | 0.0362 ± 0.0076 | 0.403 | 0.030 |
|  | Glutathione analogs: mycothiol | 0.0902 ± 0.0197 | -0.441 | 0.017 |
|  | Redox-dependent regulation of nucleus processes | 0.0592 ± 0.0087 | -0.553 | 0.002 |

**Table S5.** Environmental variables listed by sample ID

| **Sample ID** | **Longitude (▲E)** | **Latitude (▲N)** | **Elevation (m)** | **Mean annual temperature (℃)** | **Growing season temperature (℃)** |
| --- | --- | --- | --- | --- | --- |
| 1_R | 100.9 | 36.3 | 3282 | 4.7 | 13.5 |
| 10_R | 96.7 | 32.9 | 4258 | 3 | 9.9 |
| 12_R | 96.4 | 33.3 | 4284 | 0.4 | 8.4 |
| 13_R | 96 | 33.6 | 4234 | -1 | 7.6 |
| 16_R | 96.2 | 34.1 | 4366 | -2.2 | 7.2 |
| 19_R | 99.2 | 35.4 | 4162 | -0.8 | 8.8 |
| 20_R | 99.5 | 35.4 | 4089 | 0 | 9.5 |
| 21_R | 101 | 36.9 | 3066 | 3.2 | 12.7 |
| 23_R | 99.6 | 37.1 | 3241 | 0.3 | 10.6 |
| 24_R | 98.9 | 36.9 | 3297 | 2.2 | 12 |
| 25_R | 98.4 | 37 | 3130 | 3.8 | 13 |
| 26_R | 98.4 | 37.3 | 3425 | 2.7 | 12.6 |
| 27_R | 97.1 | 37.4 | 2918 | 3.9 | 14.5 |
| 28_R | 95.9 | 37.5 | 3319 | 3.4 | 14.7 |
| 29_R | 95.5 | 37.4 | 3181 | 3 | 14.5 |
| 3_R | 100.5 | 35.6 | 3320 | 2 | 11.1 |
| 30_R | 94.5 | 35.9 | 3633 | 2.1 | 11.7 |
| 31_R | 93.9 | 35.5 | 4598 | -1.2 | 8.6 |
| 32_R | 92.9 | 34.9 | 4555 | -4.9 | 5.2 |
| 34_R | 92.1 | 33.7 | 4697 | -3.4 | 5.8 |
| 36_R | 92 | 32.9 | 5228 | -2.9 | 6 |
| 37_R | 91.7 | 32.2 | 4632 | -2.3 | 6.4 |
| 40_R | 91.3 | 31.5 | 4664 | -1.2 | 7.3 |
| 43_R | 91.5 | 30.6 | 4542 | 1.7 | 9.8 |
| 44_R | 90.6 | 30.2 | 4557 | 4.2 | 11.2 |
| 5_R | 100.8 | 34.9 | 3663 | -0.3 | 9 |
| 6_R | 100.4 | 34.5 | 4250 | 0 | 8.2 |
| 8_R | 99 | 34.8 | 4505 | -2.9 | 7 |
| 9_R | 98.5 | 34.9 | 4230 | -3.5 | 6.5 |

**Table S5.** (continued)

| **Sample ID** | **Mean annual precipitation (mm)** | **Growing season precipitation (mm)** | **Potential evapotranspiration (mm/year)** | **Actual evapotranspiration (mm/year)** |
| --- | --- | --- | --- | --- |
| 1_R | 321.3 | 245.6 | 495 | 337 |
| 10_R | 488.9 | 356.4 | 428 | 428 |
| 12_R | 484.9 | 346.5 | 388 | 388 |
| 13_R | 457.9 | 326.8 | 349 | 349 |
| 16_R | 418.3 | 289.4 | 345 | 345 |
| 19_R | 330.2 | 243.1 | 373 | 345 |
| 20_R | 342.5 | 254.3 | 398 | 360 |
| 21_R | 386.8 | 269.6 | 469 | 355 |
| 23_R | 309 | 234.8 | 412 | 291 |
| 24_R | 224.5 | 182 | 450 | 240 |
| 25_R | 219.6 | 166.6 | 476 | 217 |
| 26_R | 235.9 | 177 | 461 | 219 |
| 27_R | 147.6 | 108.9 | 494 | 155 |
| 28_R | 78.2 | 61.6 | 489 | 93 |
| 29_R | 66.4 | 52.1 | 482 | 81 |
| 3_R | 404.6 | 290.1 | 439 | 401 |
| 30_R | 127.5 | 102.1 | 439 | 153 |
| 31_R | 205.4 | 153 | 359 | 202 |
| 32_R | 291.6 | 215.6 | 322 | 263 |
| 34_R | 336.6 | 261.9 | 332 | 323 |
| 36_R | 402.5 | 302.3 | 334 | 334 |
| 37_R | 436.1 | 326 | 337 | 337 |
| 40_R | 435.4 | 329.3 | 345 | 345 |
| 43_R | 509.6 | 374.9 | 410 | 410 |
| 44_R | 422.3 | 330.9 | 453 | 420 |
| 5_R | 493.6 | 347.6 | 389 | 389 |
| 6_R | 523.2 | 363.9 | 386 | 386 |
| 8_R | 377.1 | 267.7 | 344 | 344 |
| 9_R | 343.7 | 244.7 | 339 | 338 |

**Table S5.** (continued)

| **Sample ID** | **Soil moisture  (g/g of dried soil)** | **pH** | **Soil total carbon (%)** | **Soil organic carbon (%)** | **Soil total nitrogen (%)** |
| --- | --- | --- | --- | --- | --- |
| 1_R | 0.15 | 7.14 | 3.25 | 3.09 | 0.34 |
| 10_R | 1.1 | 6.97 | 15.22 | 15.21 | 1.3 |
| 12_R | 0.51 | 6.46 | 9.35 | 9.33 | 0.7 |
| 13_R | 0.71 | 7.22 | 11.41 | 11.39 | 0.88 |
| 16_R | 0.53 | 7.23 | 9.12 | 9.09 | 0.78 |
| 19_R | 0.07 | 8.4 | 3.08 | 2.07 | 0.16 |
| 20_R | 0.08 | 8.04 | 3.58 | 2.93 | 0.27 |
| 21_R | 0.08 | 8.35 | 3.87 | 3.27 | 0.33 |
| 23_R | 0.07 | 7.43 | 3.7 | 2.54 | 0.2 |
| 24_R | 0.14 | 7.91 | 3.34 | 2.12 | 0.19 |
| 25_R | 0.03 | 8.57 | 2.19 | 0.72 | 0.07 |
| 26_R | 0.06 | 8.17 | 3.09 | 2.02 | 0.25 |
| 27_R | 0.01 | 8.43 | 2.81 | 0.97 | 0.07 |
| 28_R | 0 | 8.66 | 1.85 | 0.66 | 0.02 |
| 29_R | 0.03 | 7.92 | 1.12 | 0.25 | 0.03 |
| 3_R | 0.17 | 7.8 | 5.09 | 4.85 | 0.45 |
| 30_R | 0.07 | 8.87 | 1.86 | 0.76 | 0.06 |
| 31_R | 0.05 | 8.99 | 1.33 | 0.81 | 0.07 |
| 32_R | 0.1 | 8.64 | 1.22 | 0.49 | 0.03 |
| 34_R | 0.13 | 7.98 | 2.48 | 1.92 | 0.16 |
| 36_R | 0.15 | 7.61 | 2.18 | 2.18 | 0.16 |
| 37_R | 0.05 | 8.24 | 2.47 | 1.45 | 0.09 |
| 40_R | 0.03 | 7.27 | 2.14 | 2.14 | 0.2 |
| 43_R | 0.47 | 6.03 | 11.21 | 11.2 | 0.66 |
| 44_R | 0.63 | 8.06 | 7.33 | 7.2 | 0.54 |
| 5_R | 0.6 | 7.77 | 13.57 | 13.53 | 1.11 |
| 6_R | 0.68 | 6.81 | 9.55 | 9.54 | 0.76 |
| 8_R | 1.05 | 7.8 | 11.98 | 11 | 0.94 |
| 9_R | 0.09 | 9.15 | 2.37 | 1.19 | 0.07 |

**Table S5.** (continued)

| **Sample ID** | **Soil total phosphorous (%)** | **Soil CaCO3 (%)** | **Soil carbon density (g/m**^-2^) | **Soil organic carbon density (g/m**^-2^) |
| --- | --- | --- | --- | --- |
| 1_R | 0.07 | 1.32 | 3245 | 3086.44 |
| 10_R | 0.12 | 0.08 | 15220 | 15210 |
| 12_R | 0.07 | 0.13 | 9345 | 9329.42 |
| 13_R | 0.09 | 0.19 | 11410 | 11387.07 |
| 16_R | 0.06 | 0.25 | 9120 | 9089.73 |
| 19_R | 0.06 | 8.46 | 3080 | 2065.12 |
| 20_R | 0.06 | 5.35 | 3575 | 2933.11 |
| 21_R | 0.07 | 4.95 | 3865 | 3270.46 |
| 23_R | 0.06 | 9.66 | 3695 | 2535.59 |
| 24_R | 0.07 | 10.13 | 3340 | 2124.49 |
| 25_R | 0.07 | 12.29 | 2190 | 715 |
| 26_R | 0.09 | 5.28 | 3085 | 2016.61 |
| 27_R | 0.07 | 15.31 | 2805 | 967.74 |
| 28_R | 0.06 | 9.92 | 1850 | 659.62 |
| 29_R | 0.04 | 7.27 | 1120 | 247.14 |
| 3_R | 0.08 | 1.96 | 5090 | 4854.52 |
| 30_R | 0.06 | 9.22 | 1863.33 | 757.53 |
| 31_R | 0.04 | 4.28 | 1325 | 811.77 |
| 32_R | 0.02 | 6.05 | 1215 | 489.34 |
| 34_R | 0.04 | 4.71 | 2480 | 1915.16 |
| 36_R | 0.05 | 0 | 2175 | 2175 |
| 37_R | 0.02 | 8.49 | 2465 | 1446.21 |
| 40_R | 0.04 | 0 | 2135 | 2135 |
| 43_R | 0.07 | 0.04 | 11205 | 11199.98 |
| 44_R | 0.07 | 1.07 | 7325 | 7197 |
| 5_R | 0.11 | 0.29 | 13565 | 13530.09 |
| 6_R | 0.09 | 0.12 | 9550 | 9535.04 |
| 8_R | 0.08 | 8.15 | 11980 | 11001.65 |
| 9_R | 0.05 | 9.77 | 2365 | 1192.66 |

**Table S5.** (continued)

| **Sample ID** | **Soil total nitrogen density (g/m**^-2^) | **Soil total phosphorous density (g/m**^-2^) | **Soil CaCO3 density (g/m**^-2^) | **Soil C:N ratio** |
| --- | --- | --- | --- | --- |
| 1_R | 340 | 65.56 | 738.93 | 9.54 |
| 10_R | 1295 | 119.62 | 18.97 | 11.75 |
| 12_R | 695 | 73.66 | 45.34 | 13.45 |
| 13_R | 880 | 86.8 | 76.78 | 12.97 |
| 16_R | 775 | 63.9 | 88.53 | 11.77 |
| 19_R | 155 | 64.41 | 4378.51 | 19.87 |
| 20_R | 265 | 60.27 | 2651.61 | 13.49 |
| 21_R | 330 | 67.71 | 3020.83 | 11.71 |
| 23_R | 200 | 57.26 | 5215.11 | 18.48 |
| 24_R | 190 | 67.43 | 5913.64 | 17.58 |
| 25_R | 65 | 70.69 | 7553.41 | 33.69 |
| 26_R | 250 | 91.8 | 2742.92 | 12.34 |
| 27_R | 70 | 68.27 | 8710.38 | 40.07 |
| 28_R | 20 | 61.19 | 7346.49 | 75 |
| 29_R | 30 | 40.01 | 4938.94 | 37.33 |
| 3_R | 445 | 77.06 | 842.38 | 11.44 |
| 30_R | 56.67 | 61.11 | 6920.21 | 32.88 |
| 31_R | 70 | 42.85 | 2300.28 | 18.93 |
| 32_R | 30 | 21.18 | 4075.41 | 40.5 |
| 34_R | 160 | 40.68 | 2821.75 | 15.5 |
| 36_R | 160 | 46.71 | 0 | 13.59 |
| 37_R | 85 | 21.25 | 4578.88 | 29 |
| 40_R | 195 | 37.54 | 0 | 10.95 |
| 43_R | 660 | 73.43 | 16.88 | 16.98 |
| 44_R | 535 | 73.38 | 578.7 | 13.69 |
| 5_R | 1110 | 106.95 | 111.32 | 12.22 |
| 6_R | 755 | 88.88 | 32.78 | 12.65 |
| 8_R | 940 | 79.55 | 1767.97 | 12.74 |
| 9_R | 65 | 49.77 | 4969.57 | 36.38 |

**Table S5.** (continued)

| **Sample ID** | **Soil N:P ratio** | **Dissolved organic carbon (mg/kg of dried soil)** | **Dissolved total nitrogen (mg/kg of dried soil)** |
| --- | --- | --- | --- |
| 1_R | 5.19 | 98.85 | 55.61 |
| 10_R | 10.83 | 353.67 | 232.25 |
| 12_R | 9.44 | 610.07 | 109.57 |
| 13_R | 10.14 | 410.94 | 83.86 |
| 16_R | 12.13 | 423.81 | 69.35 |
| 19_R | 2.41 | 112.95 | 21 |
| 20_R | 4.4 | 201.74 | 23.82 |
| 21_R | 4.87 | 148.65 | 16.83 |
| 23_R | 3.49 | 259.34 | 17.81 |
| 24_R | 2.82 | 232.48 | 62.13 |
| 25_R | 0.92 | 97.27 | 10.72 |
| 26_R | 2.72 | 202.72 | 28.16 |
| 27_R | 1.03 | 327.28 | 39.96 |
| 28_R | 0.33 | 311.91 | 12.11 |
| 29_R | 0.75 | 175.43 | 13.24 |
| 3_R | 5.77 | 242.77 | 73.32 |
| 30_R | 0.93 | 142.97 | 12.29 |
| 31_R | 1.63 | 146.63 | 8.01 |
| 32_R | 1.42 | 243.52 | 28.87 |
| 34_R | 3.93 | 261.12 | 34.62 |
| 36_R | 3.43 | 190.71 | 20.06 |
| 37_R | 4 | 151.68 | 12.48 |
| 40_R | 5.19 | 163.21 | 13.91 |
| 43_R | 8.99 | 896.4 | 63.22 |
| 44_R | 7.29 | 347.12 | 83.49 |
| 5_R | 10.38 | 320.42 | 190.91 |
| 6_R | 8.49 | 78.01 | 120.91 |
| 8_R | 11.82 | 84 | 98.56 |
| 9_R | 1.31 | 36.27 | 9.05 |

**Table S5.** (continued)

| **Sample ID** | **Dissolved organic nitrogen (mg/kg of dried soil)** | **Nitrate nitrogen (g/kg of dried soil)** | **Ammonium nitrogen (g/kg or dried soil)** |
| --- | --- | --- | --- |
| 1_R | 18.61 | 25.74 | 11.26 |
| 10_R | 101.65 | 106.1 | 24.51 |
| 12_R | 66.77 | 22.92 | 19.88 |
| 13_R | 49.69 | 5.03 | 29.14 |
| 16_R | 40.31 | 16.3 | 12.74 |
| 19_R | 12.68 | 3.76 | 4.56 |
| 20_R | 10.09 | 8.8 | 4.94 |
| 21_R | 4.7 | 6.78 | 5.36 |
| 23_R | 6.29 | 5.63 | 5.89 |
| 24_R | 9.69 | 36.26 | 16.19 |
| 25_R | 0.99 | 1.69 | 8.04 |
| 26_R | 6.76 | 10.76 | 10.64 |
| 27_R | 14.12 | 16.65 | 9.19 |
| 28_R | 1.74 | 2.88 | 7.49 |
| 29_R | 3.57 | 1.05 | 8.61 |
| 3_R | 32.29 | 31.68 | 9.35 |
| 30_R | 1.27 | 4.01 | 7.01 |
| 31_R | 1.16 | 0.29 | 6.56 |
| 32_R | 2.3 | 5.28 | 21.29 |
| 34_R | 8.15 | 17.85 | 8.62 |
| 36_R | 4.58 | 8.54 | 6.93 |
| 37_R | 3.18 | 2.32 | 6.97 |
| 40_R | 6.17 | 0 | 7.74 |
| 43_R | 38.13 | 0.33 | 24.75 |
| 44_R | 15.66 | 55.54 | 12.28 |
| 5_R | 76.41 | 92.99 | 21.51 |
| 6_R | 31.64 | 62.4 | 26.87 |
| 8_R | 7.84 | 68.6 | 22.12 |
| 9_R | 3.02 | 0.5 | 5.53 |

**Table S5.** (continued)

| **Sample ID** | **Total available nitrogen (sum of ammonium, nitrate, and dissolved organic nitrogen) (mg/kg of dried soil)** | **Soil bulk density (g/m^-3^)** | **Above ground biomass (g/m^-2^)** |
| --- | --- | --- | --- |
| 1_R | 55.61 | 1.13 | 36.65 |
| 10_R | 232.25 | 0.45 | 63.91 |
| 12_R | 109.57 | 0.69 | 67.49 |
| 13_R | 83.86 | 0.77 | 44.45 |
| 16_R | 69.35 | 0.93 | 78.02 |
| 19_R | 21 | 1.05 | 40.01 |
| 20_R | 23.82 | 0.99 | 28.49 |
| 21_R | 16.83 | 1.19 | 44.45 |
| 23_R | 17.81 | 1.09 | 110.07 |
| 24_R | 62.13 | 1.22 | 218.7 |
| 25_R | 10.72 | 1.23 | 87.08 |
| 26_R | 28.16 | 0.99 | 175 |
| 27_R | 39.96 | 1.25 | 187.12 |
| 28_R | 12.11 | 1.48 | 161.17 |
| 29_R | 13.24 | 1.44 | 59.83 |
| 3_R | 73.32 | 0.84 | 211.66 |
| 30_R | 12.29 | 1.59 | 105.74 |
| 31_R | 8.01 | 1.15 | 48.05 |
| 32_R | 28.87 | 1.4 | 248.16 |
| 34_R | 34.62 | 1.21 | 118.79 |
| 36_R | 20.06 | 0.52 | 50.11 |
| 37_R | 12.48 | 1.22 | 66.13 |
| 40_R | 13.91 | 1.16 | 54.71 |
| 43_R | 63.22 | 0.88 | 41.39 |
| 44_R | 83.49 | 1.08 | 283.09 |
| 5_R | 190.91 | 0.6 | 225.05 |
| 6_R | 120.91 | 0.63 | 123.85 |
| 8_R | 98.56 | 0.44 | 44.81 |
| 9_R | 9.05 | 1 | 54.13 |

**Table S5.** (continued)

| **Sample ID** | **Root biomass at 5cm soil depth (g/m**^-2^) | **% total carbon in aboveground biomass** | **% total nitrogen in aboveground biomass** |
| --- | --- | --- | --- |
| 1_R | 86.76 | 45.63 | 2.65 |
| 10_R | 1859.92 | 38.42 | 1.78 |
| 12_R | 1836.22 | 44.14 | 1.91 |
| 13_R | 1272.86 | 40.9 | 2.74 |
| 16_R | 746.5 | 38.16 | 2.02 |
| 19_R | 402.67 | 42.79 | 1.19 |
| 20_R | 428.44 | 44.24 | 1.8 |
| 21_R | 197.9 | 41.22 | 1.77 |
| 23_R | 106.52 | 45.61 | 1.54 |
| 24_R | 822.17 | 43.25 | 2.49 |
| 25_R | 197.32 | 38.92 | 1.38 |
| 26_R | 136.79 | 43.09 | 1.46 |
| 27_R | 14.37 | 42.96 | 1.12 |
| 28_R | 1.68 | 47.96 | 0.65 |
| 29_R | 1.87 | 41.05 | 1.23 |
| 3_R | 393.62 | 43.25 | 1.9 |
| 30_R | 19.96 | 41.5 | 1.6 |
| 31_R | 1268.63 | 37.6 | 1.97 |
| 32_R | 558.31 | 38.88 | 1.79 |
| 34_R | 824.27 | 35.42 | 1.43 |
| 36_R | 783.63 | 27.88 | 1.52 |
| 37_R | 2071.37 | 42.18 | 1.69 |
| 40_R | 3004.02 | 38 | 1.73 |
| 43_R | 2308.09 | 42.87 | 1.89 |
| 44_R | 819.99 | 44.33 | 1.61 |
| 5_R | 812.9 | 43.18 | 1.94 |
| 6_R | 575.16 | 42.73 | 2.07 |
| 8_R | 749.62 | 37.39 | 1.95 |
| 9_R | 155.5 | 36.21 | 1.49 |

**Table S5.** (continued)

| **Sample ID** | **% total phosphorous in aboveground biomass** | **C:N ratio in aboveground biomass** | **P:N ratio in aboveground biomass** | **Vegetation type** |
| --- | --- | --- | --- | --- |
| 1_R | 0.14 | 17.22 | 19.26 | alpine steppe |
| 10_R | 0.16 | 21.58 | 10.93 | alpine meadow |
| 12_R | 0.18 | 23.11 | 10.37 | alpine meadow |
| 13_R | 0.24 | 14.93 | 11.52 | alpine meadow |
| 16_R | 0.18 | 18.89 | 10.96 | alpine meadow |
| 19_R | 0.1 | 35.96 | 12.08 | alpine steppe |
| 20_R | 0.14 | 24.58 | 12.83 | alpine steppe |
| 21_R | 0.1 | 23.29 | 17.57 | alpine steppe |
| 23_R | 0.11 | 29.62 | 13.42 | alpine steppe |
| 24_R | 0.12 | 17.37 | 20.08 | alpine steppe |
| 25_R | 0.1 | 28.2 | 13.72 | desert steppe |
| 26_R | 0.12 | 29.51 | 12.03 | alpine steppe |
| 27_R | 0.09 | 38.36 | 12.12 | desert steppe |
| 28_R | 0.08 | 73.78 | 8.04 | desert steppe |
| 29_R | 0.09 | 33.37 | 13.52 | desert steppe |
| 3_R | 0.2 | 22.76 | 9.7 | alpine meadow |
| 30_R | 0.09 | 25.94 | 17.53 | desert steppe |
| 31_R | 0.14 | 19.09 | 13.76 | alpine steppe |
| 32_R | 0.15 | 21.72 | 12.16 | alpine meadow |
| 34_R | 0.16 | 24.77 | 9.02 | alpine steppe |
| 36_R | 0.18 | 18.34 | 8.4 | alpine meadow |
| 37_R | 0.18 | 24.96 | 9.63 | alpine meadow |
| 40_R | 0.11 | 21.97 | 15.49 | alpine meadow |
| 43_R | 0.12 | 22.68 | 15.31 | alpine meadow |
| 44_R | 0.11 | 27.53 | 14 | alpine meadow |
| 5_R | 0.17 | 22.26 | 11.29 | alpine meadow |
| 6_R | 0.15 | 20.64 | 13.89 | alpine meadow |
| 8_R | 0.12 | 19.17 | 15.98 | alpine meadow |
| 9_R | 0.14 | 24.3 | 10.96 | alpine steppe |

**Table S6.** Averaged environmental variables for each vegetation type (average ± standard deviation)

| Measured environmental factors | Alpine meadow | Alpine steppe | Desert steppe |
| --- | --- | --- | --- |
| Elevation (m) | 4361 ± 453 | 3809 ± 610 | 3236 ± 265 |
| Mean annual temperature (℃) | -0.5 ± 2.5 | 0.4 ± 2.8 | 3.2 ± 0.7 |
| Growing season temperature (℃) | 8.2 ± 1.8 | 10.1 ± 2.7 | 13.7 ± 1.3 |
| Mean annual precipitation (mm) | 439 ± 61.3 | 303.6 ± 60.2 | 127.9 ± 61.3 |
| Growing season precipitation (mm) | 319.1 ± 42.8 | 226.6 ± 40.5 | 98.3 ± 45.5 |
| Potential evapotranspiration (mm/year) | 376 ± 43 | 409 ± 58 | 476 ± 22 |
| Actual evapotranspiration (mm/year) | 367 ± 44 | 301 ± 59 | 140 ± 55 |
| Soil moisture (g/g of dried soil) | 0.48 ± 0.35 | 0.09 ± 0.04 | 0.03 ± 0.03 |
| pH | 7.42 ± 0.71 | 8.16 ± 0.62 | 8.49 ± 0.36 |
| Soil total carbon (%) | 7.99 ± 4.64 | 3.01 ± 0.76 | 1.97 ± 0.61 |
| Soil organic carbon (%) | 7.76 ± 4.77 | 2.2 ± 0.79 | 0.67 ± 0.26 |
| Soil total nitrogen (%) | 0.61 ± 0.39 | 0.2 ± 0.09 | 0.05 ± 0.02 |
| Soil total phosphorous (%) | 0.07 ± 0.03 | 0.06 ± 0.02 | 0.06 ± 0.01 |
| Soil CaCO3 (%) | 1.92 ± 3.15 | 6.39 ± 2.94 | 10.8 ± 3.09 |
| Soil carbon density (g/m^-2^) | 7986 ± 4643 | 3006 ± 762 | 1966 ± 611 |
| Soil organic carbon density  (g/m^-2^) | 7755.72 ± 4771.66 | 2195.14 ± 793.17 | 669.41 ± 263.27 |
| Soil total nitrogen density (g/m^-2^) | 611 ± 390 | 203 ± 95 | 48 ± 22 |
| Soil total phosphorous density (g/m^-2^) | 69.28 ± 29.13 | 60.77 ± 14.72 | 60.25 ± 12.09 |
| Soil CaCO3 density (g/m^-2^) | 873.85 ± 1546.32 | 3475.32 ± 1589.48 | 7093.89 ± 1374.95 |
| Soil C:N ratio | 15.98 ± 8.39 | 17.38 ± 7.52 | 43.79 ± 17.68 |
| Soil N:P ratio | 7.81 ± 3.35 | 3.28 ± 1.32 | 0.79 ± 0.28 |
| Dissolved organic carbon  (mg/kg of dried soil) | 322.6 ± 220.65 | 170.08 ± 74.14 | 210.97 ± 103.12 |
| Dissolved total nitrogen  (mg/kg of dried soil) | 85.77 ± 64.16 | 27.7 ± 18.33 | 17.66 ± 12.5 |
| Dissolved organic nitrogen (mg/kg of dried soil) | 34.04 ± 30.85 | 8.12 ± 5.04 | 4.34 ± 5.56 |
| Nitrate nitrogen  (g/kg of dried soil) | 34.15 ± 36.37 | 11.64 ± 11.68 | 5.26 ± 6.47 |
| Ammonium nitrogen  (g/kg or dried soil) | 17.58 ± 7.93 | 7.96 ± 3.74 | 8.07 ± 0.87 |
| Total available nitrogen (sum of ammonium, nitrate, and dissolved organic nitrogen)  (mg/kg of dried soil) | 85.77 ± 64.16 | 27.7 ± 18.33 | 17.66 ± 12.5 |

**Table S6.** (continued)

| Measured environmental factors | Alpine meadow | Alpine steppe | Desert steppe |
| --- | --- | --- | --- |
| Soil bulk density (g/m^-3^) | 0.83 ± 0.3 | 1.1 ± 0.09 | 1.4 ± 0.15 |
| Above ground biomass (g/m^-2^) | 114.49 ± 87.4 | 87.43 ± 66 | 120.19 ± 52.7 |
| Root biomass at 5cm soil depth (g/m^-2^) | 1270.87 ± 801.22 | 442.97 ± 401.28 | 47.04 ± 84.38 |
| % total carbon in aboveground biomass | 40.17 ± 4.32 | 41.51 ± 3.79 | 42.48 ± 3.39 |
| % total nitrogen in aboveground biomass | 1.9 ± 0.29 | 1.78 ± 0.47 | 1.2 ± 0.35 |
| % total phosphorous in aboveground biomass | 0.16 ± 0.04 | 0.13 ± 0.02 | 0.09 ± 0.01 |
| C:N ratio in aboveground biomass | 21.47 ± 3.05 | 24.57 ± 5.94 | 39.93 ± 19.52 |
| P:N ratio in aboveground biomass | 12.12 ± 2.42 | 14.1 ± 3.66 | 12.99 ± 3.42 |
